# National Trends of Arrhythmia Hospitalizations and Comorbid Alcohol Use Disorders in the United States

**DOI:** 10.7759/cureus.8835

**Published:** 2020-06-25

**Authors:** Virendrasinh Ravat, Temitope Ajibawo, Tarun Parvataneni, Kristal N Pereira, Ting Yu Yen, Rikinkumar S Patel

**Affiliations:** 1 Internal Medicine, Krishna Institute of Medical Sciences, Karad, IND; 2 Internal Medicine, Brookdale University Hospital Medical Center, New York City, USA; 3 Psychiatry, Siddavanahalli Nijalingappa Medical College and HSK Hospital and Research Centre, Bagalkot, IND; 4 Internal Medicine, Terna Medical College, Mumbai, IND; 5 Medicine, Poznan University of Medical Sciences, Poznan, POL; 6 Psychiatry, Griffin Memorial Hospital, Norman, USA

**Keywords:** ventricular arrhythmia, arrhythmia, mortality, alcohol dependence, alcohol misuse, alcohol use, national trend, hospital epidemiology

## Abstract

Objective

To study the trends of arrhythmia hospitalizations with comorbid alcohol use disorders (AUDs) in terms of demographic characteristics and inpatient outcomes.

Methods

We used the Nationwide Inpatient Sample (NIS) data from 2010 to 2014 and included 570,556 arrhythmia inpatients (age, 15-54 years), and 55,730 inpatients had comorbid AUD. We used the linear-by-linear association test for measuring the differences in demographics, comorbidities, and hospital outcomes over the study period of 2010 to 2014, and the analysis of variance (ANOVA) for measuring the changes seen in the length of stay (LOS) and total charges.

Results

Arrhythmia inpatients with AUD were majorly males (85.9%), and older-age adults (45 to 54 years, 68%). Hypertension (52.2%), tobacco abuse (42.3%), and elevated cholesterol and lipids (22.6%) were the most prevalent comorbidities in the study population. There was a statistically significant increasing trend in arrhythmia inpatients with AUD with comorbid diabetes, hypertension, and obesity over the five-year period. In-hospital mortality had a variable trend from 1.1% in 2010 to 1.3% in 2014, but there was a statistically non-significant difference in the trend (P = 0.418). Mean LOS was three days with statistically no significant change during the study period (P = 0.080), whereas total charges have been increasing significantly (P <0.001), averaging $37,473 per hospitalization.

Conclusion

The prevalence trend of arrhythmia hospitalizations with comorbid AUD is increasing in the United States population, and is majorly seen in older-age men. Overall, in-hospital mortality in arrhythmia inpatients with comorbid AUD was 1.4%. So, this necessitates the development of an integrated clinical care model for early diagnosis and management of alcohol abuse and dependence in order to improve the arrhythmia patient outcomes and quality of life.

## Introduction

Holiday heart syndrome (HHS) is an acute cardiac rhythm and/or conduction disturbance associated with heavy ethanol consumption in a person without other clinical evidence of heart disease. The initial recognition of HHS was a result of a study evaluating 32 dysrhythmic episodes in 24 hospitalized patients who consumed alcohol heavily and regularly; in addition, they took part in a weekend or holiday binge drinking. In their series, the most common cardiac rhythm disturbances were supraventricular tachyarrhythmias (SVT) and atrial fibrillation (AF). Typically, this resolved rapidly with spontaneous recovery during subsequent abstinence from alcohol use [1]. Estimates of the prevalence of AF in the United States (US) ranged from 2.7 to 6.1 million in 2010 and AF prevalence is estimated to rise to 12.1 million in 2030 [2-4].

According to the 2018 National Survey on Drug Use and health (NSDUH), 14.4 million adults (5.8% of this age group >18 years) had AUD, which includes 9.2 million men and 5.3 million women. Furthermore, an estimated 401,000 adolescents (1.6% of this age group 12-17 years) had AUD with 173,000 males and 227,000 females [5]. Several mechanisms are theorized to be responsible for the arrhythmogenicity of alcohol. They may be characterized into two broad groups: direct effects on the myocardium and alcohol's effect on traditional risk factors for AF. On the atrial myocardium, alcohol causes an autonomic nervous system imbalance. Alcohol increases sympathetic nervous system (SNS) activity (and its related increased secretion of epinephrine and norepinephrine), with resultant effects including an increased release of calcium into the myocytes from the sarcoplasmic reticulum [6,7]. Increased SNS activity is further evidenced by a marked increase in the incidence of sinus tachycardia and reduced respiratory sinus arrhythmia during acute alcohol intoxication. Consequently, the parasympathetic nervous system (PNS) is activated as well, with an increased intermittent vagal tone, which has been shown to also shorten the atrial refractory period and precipitate AF [8,9]. The risk of AF persists into the "hangover" and/or withdrawal phase, which corresponds with an increased sympathetic tone. 

We utilized the National Inpatient Sample (NIS) from the US hospitals to study the national trends of arrhythmia-related hospitalizations with comorbid alcohol use disorders (AUDs) in terms of demographic characteristics and inpatient outcomes, including the severity of illness, length of stay (LOS) and total charges, and in-hospital mortality.

## Materials and methods

Data source

We conducted a cross-sectional data analysis of the NIS from January 2010 to December 2014. This dataset provides discharge inpatient data from 4,400 community-based hospitals across 44 states in the United States. Diagnostic information in the NIS is detected using the International Classification of Diseases, ninth edition (ICD-9) codes [10].

Inclusion criteria and outcome variables

Our study population included patients (age 15 to 54 years) with a primary ICD-9 discharge diagnosis for arrhythmia (427.0-427.2, 427.31, 427.32, 427.60, 427.61, 427.69, 427.81, 427.89, 427.9, 785.0 or 785.1) and comorbid AUD using the ICD-9 codes 291.0-291.3, 291.5, 291.8, 291.81, 281.82, 291.89, 291.9, 303.00-303.93, or 305.00-305.03.

Demographic characteristics included in the data analysis were age group (15-24, 25-34, 35-44, 45-54 years), sex (male or female), and race (Caucasian, African American, Hispanic, or others) [10]. The risk factors for arrhythmia were included after reviewing past literature and detected in the NIS using ICD-9 codes for diabetes, hypertension, obesity, atherosclerosis, elevated cholesterol and lipids, and tobacco abuse.

We measured the differences in hospital outcomes including the severity of illness (i.e., loss of body functions stratified by minor, moderate and major) and in-hospital mortality (number of inpatient deaths and were all-cause). LOS is considered as the total number of nights the patient stayed in the hospital for arrhythmia, and total charges during inpatient management do not include professional fees or non-covered charges [10].

Statistical analysis

In our study, we conducted the data analysis using the Statistical Package for the Social Sciences (SPSS), version 26 (IBM Corporation, Armonk, NY). We used the linear-by-linear association test for measuring the differences in demographics, comorbidities, and inpatient outcomes over the study period of 2010 to 2014, and the analysis of variance (ANOVA) for measuring the changes seen in LOS and total charges. A P-value lesser than 0.01 was considered for statistical significance.

Ethical approval

Individual identifiers were used to protect the patient's health information; we do not require approval from an institutional review board as the NIS is a de-identified data [10].

## Results

We analyzed 570,556 arrhythmias inpatients from 2010 to 2014, and 55,730 had comorbid AUD (9.8%). There was a variable trend in arrhythmia inpatients with AUD with 10,436 (in 2010) and increased to 11,355-11,680 between 2011 and 2013 and then dropped to 10,900 (in 2014). But by prevalence, there was a statistically significant increase in comorbid AUD from 8.5% in 2010 to 10.8% in 2014 (P < 0.001).

Arrhythmia inpatients with AUD were majorly males (85.9%), and older-age adults 45 to 54 years (68%). About three-fourth of them were Caucasian (69%) followed by African American (16.3%), Hispanic (10.2%), and other races (4.5%). There was a statistically non-significant difference in the age group, sex, and race during the study period (2010 to 2014).

Hypertension (52.2%), tobacco abuse (42.3%), and elevated cholesterol and lipids (22.6%) were the most prevalent comorbidities in the study population. There was a statistically significant increasing trend in arrhythmia with AUD patients with diabetes, hypertension, and obesity over the five-year period. Though tobacco abuse was prevalent but over the years, there was a non-significant change (P = 0.900). The demographic and comorbidities trends in arrhythmia inpatients with AUD are shown in Table 1.

**Table 1:** Demographic trends of arrhythmia inpatients with alcohol use disorders AUD, alcohol use disorder

Variable	2010	2011	2012	2013	2014	Total	P-value
Arrhythmia inpatients	122,592	122,749	116,610	108,050	100,555	570,556	-
Arrhythmia inpatients with AUD	10,436	11,359	11,680	11,355	10,900	55,730	-
Prevalence	8.5	9.3	10.0	10.5	10.8	9.8	<0.001
Age at admission, in %							
15 – 24 years	2.8	3.0	3.0	2.4	2.0	2.6	0.031
25 – 34 years	9.8	11.0	10.5	10.1	11.2	10.5	
35 – 44 years	20.6	19.8	18.4	18.1	17.4	18.9	
45 – 54 years	66.8	66.1	68.1	69.4	69.4	68.0	
Sex, in %							
Male	86.1	85.2	85.5	86.4	86.4	85.9	0.394
Female	13.9	14.8	14.5	13.6	13.6	14.1	
Race, in %							
Caucasian	68.3	70.7	69.0	68.6	68.4	69.0	0.385
African American	16.8	15.8	16.4	16.0	16.5	16.3	
Hispanic	10.2	9.5	10.2	10.6	10.4	10.2	
Other	4.7	4.1	4.4	4.7	4.7	4.5	
Comorbid risk factors, in %							
Diabetes	10.1	10.9	12.6	12.9	11.9	11.7	0.010
Hypertension	50.0	48.9	53.2	53.1	55.5	52.2	<0.001
Obesity	13.7	14.6	16.1	17.2	18.9	16.1	<0.001
Atherosclerosis	1.3	1.2	1.7	1.5	1.6	1.5	0.190
Elevated cholesterol and lipids	22.2	21.0	23.5	22.9	23.3	22.6	0.123
Tobacco abuse	42.6	41.6	42.7	42.0	42.6	42.3	0.900

The majority of the arrhythmia inpatients with AUD were hospitalized on non-elective admission (94.9%). There was a statistically non-significant difference in the trend by the severity of illness from 2010 to 2014 (P = 0.646). Also, in-hospital mortality had a variable trend from 1.1% in 2010 and 1.3% in 2014, but there was a statistically non-significant difference in the trend (P = 0.418). Mean LOS was three days with statistically no significant change during the study period (P = 0.080), whereas total charges have been increasing significantly (P < 0.001), averaging $37,473 per hospitalization. Trends in inpatient outcomes are shown in Table 2.

**Table 2:** Inpatient outcome trends of arrhythmia inpatients with alcohol use disorders

Variable	2010	2011	2012	2013	2014	Total	P-value
Admission type, in %							
Non-elective	93.9	93.4	95.7	95.5	95.9	94.9	<0.001
Elective	6.1	6.6	4.3	4.5	4.1	5.1	
Severity of illness, in %							
Minor	28.0	27.2	28.6	27.9	25.6	27.5	0.646
Moderate	39.7	36.9	45.1	43.1	41.7	41.3	
Major	32.3	35.9	26.2	29.0	32.7	31.2	
In-hospital mortality, in %							
Inpatient deaths	1.1	1.4	1.7	1.6	1.3	1.4	0.418
Other outcomes							
Mean length of stay, in days	3.4	3.4	3.4	3.4	3.8	3.5	0.080
Mean total charges, in $	33,146	37,107	35,659	37,839	43,632	37,473	<0.001

## Discussion

The prevalence of comorbid AUD in arrhythmias inpatients during the five-year study period was 9.8%, with a statistically significant increase in its prevalence from 8.5% in 2010 to 10.8% in 2014. During 2012 to 2013, the US prevalence for 12-month and lifetime AUD among the adult population were 13.9% and 29.1%, respectively [11]. In our study, arrhythmia inpatients with AUD were majorly older-age men aged 45 to 54 years. The prevalence AF is higher in men, and after the sixth decade of life, it doubles approximately every 10 years, from 0.5% at age 50 to 59 years to almost 9% at age 80 to 89 years [12].

Hypertension, tobacco abuse, and hypercholesterolemia and hyperlipidemia were the most prevalent comorbidities, and comorbid diabetes, hypertension, and obesity had an increasing trend in arrhythmia inpatients with AUD. Alcohol may be responsible for 16% of hypertensive disease with the incidence of hypertension increased by 40% if consuming >14 drinks per week [13]. A multinational meta‐analysis showed a linear relationship between alcohol and blood pressure, with a relative risk for hypertension of 1.7 for 50 g ethanol per day and 2.5 at 100 g ethanol per day [14]. Drinking more than 21 standard drinks per week and binge drinking can increase BMI, waist circumference, and waist-to-hip ratio [15]. A recent population-based study with 3,600 participants suggested that persistent uncontrolled diabetes may pose a cumulative risk of AF initiation increased by 1.4 times compared to non-diabetic. This finding suggests that strict long-term glucose control may play a significant role in decreasing the incidence of new-onset AF [16].

In our study, in-hospital mortality had a variable and statistically non-significant trend from 1.1% In 2010 and 1.3% in 2014. The association between heavy alcohol consumption and increased risk of cardiac arrhythmias exists with a significant risk associated with more than three drinks per day on average (≥36 g/day of pure ethanol) as per the Framingham study [17]. Cardy et al. found atrial and ventricular slowing of conduction (prolongation of P and QRS waves) after acute ingestion of alcohol [18]. The mortality risk may decline threefold if a person with a usual alcohol intake of 96 g/day cuts down to 36 g/day compared with a constant daily intake of 60 g/day [19]. In a long‐term prospective cohort study, 15% of heavy drinkers (50 g/day or more daily) died after 10 years and 39.1% after 20 years, compared to the low-volume drinkers (<50 g/day per drinking, less than once per month) [20]. Observational and prospective studies have consistently shown a lower risk of all‐cause and cardiovascular mortality in people with low levels of alcohol consumption when compared with abstainers, with the highest risks occurring in people with high levels of consumption. Compared with lifetime abstainers, individuals who were classified as light or moderate consumers were at reduced risk of all‐cause mortality and cardiovascular mortality, but that risk increased significantly with heavy alcohol consumption [21-24]. Also, an inpatient study in arrhythmia patients found that AUD is an independent risk factor that increases the mortality by 72% and this risk is higher in females and older adults [25].

Few limitations in our study include the underreporting of AUD, due to inconsistency in administrative data based on ICD-9 codes, including diagnostic codes during patient billing. There were few recent studies that found cannabis is an independent cardiovascular risk factor for arrhythmias and myocardial infarction, but we were not able to study the trend of comorbid substance use including cannabis abuse or dependence [26-29]. Some of the major strengths our study includes nationwide data analysis covering 44 states across the United States; moreover, our results have appropriate external validity to the American population and a strong methodology including that statistical models to evaluate the national trends of arrhythmia inpatients with AUD.

## Conclusions

The prevalence trend of arrhythmia hospitalizations with comorbid AUD is increasing in the US population, and is majorly seen in older-age men. Arrhythmia inpatients with AUD had a significant rising trend in comorbid diabetes, hypertension, and obesity due to which there was a rising trend in total hospitalization charges over the five-year study period. Overall, in-hospital mortality in arrhythmia inpatients with comorbid AUD was 1.4%. So, this necessitates the development of an integrated clinical care model for early diagnosis and management of alcohol abuse and dependence in order to improve the arrhythmia patient outcomes and quality of life.
